# A Novel Approach to Increase Attention during Mirror Therapy among Stroke Patients: A Video-Based Behavioral Analysis

**DOI:** 10.3390/brainsci12030297

**Published:** 2022-02-22

**Authors:** Sungbae Jo, Hyunjin Kim, Changho Song

**Affiliations:** Department of Physical Therapy, College of Health Science, Sahmyook University, Seoul 01795, Korea; rew277@gmail.com (S.J.); kimhj4069@hanmail.net (H.K.)

**Keywords:** stroke, mirror movement therapy, rehabilitation

## Abstract

Stroke is a major cause of disability and an evident rehabilitation strategy is crucial. Mirror therapy (MT) is one of the popular rehabilitation methods that is known to be effective as the patients benefit from the mirror illusion. However, the patient’s attention to the mirror illusion during treatment is unclear. Therefore, the present study assesses the duration and frequency of the mirror gaze, distraction, and preparation of sixteen stroke patients during two MT methods using a behavioral coding software. During the 30 min treatment, the total mirror gaze duration during conventional bilateral MT (BMT) was 564.04 s, while it was 1482.45 s in unilateral MT using a screen (UMT). The total distracted time was 945.61 s in BMT, while it was only 162.03 s in UMT. The total preparatory duration was 290.35 s in BMT and 155.53 s in UMT. The total number of distracted bouts were 136.45 in BMT, while it was 73.38 in UMT. The total number of preparatory bouts were 18.42 in BMT and 9.56 in UMT. The average times of gaze duration per bout were 5.52 s in BMT and 21.81 s in UMT. The average times of distraction per bout were 9.22 s in BMT and 3.00 s in UMT. The total number of mirror gaze bouts and average time of preparation per bout did not present a statistical significance in the comparisons of the two methods. This study assesses two methods of MT using observational coding software to evaluate the duration and frequency of the mirror gaze during treatment. The results suggest that UMT may be an alternative option to provide MT for stroke patients to increase their attention towards the mirror.

## 1. Introduction

Stroke is one of the primary causes of prolonged disability [1], and nearly 80% of survivors present with impairments in the extremities immediately after the onset [2]. Stroke characteristics include focal cerebral ischemia or hemorrhage, causing the abrupt loss of brain function that results in neurological impairments [3]. In addition, stroke can lead to other severe complications, such as pain [4], visuospatial neglect [5], and attention deficits [6], which can negatively affect functional recovery and reduce the quality of life [4,7,8,9,10,11]. Although statistical data suggests that the overall stroke incidence is declining, the lifetime risk of stroke is increasing globally, owing to aging populations and the accumulation of risk factors [12]. According to recent statistics, global deaths related to cardiovascular disease increased by 21.1% in 2017 compared to 2007 [12]. Therefore, there is a great need for efficient rehabilitation strategies to reduce physical impairments and the overall impact of stroke [13].

Currently, there are several rehabilitation strategies, including mirror therapy (MT) [14,15], constraint-induced movement therapy [16,17], action observation therapy [18], robot-assisted therapy [19], virtual reality-based training [20], electromyographic feedback [21], and acupuncture [22]. MT has been a promising strategy that has gained popularity for its simple, cheap, and manipulative characteristics [23]. This method was first described by Ramachandra et al. [24] who discovered that the use of mirrors could mediate phantom limbs in amputees. Later, Altschuler et al. [25] applied MT to hemiplegia and found an improvement, indicating the potential of utilizing MT in stroke rehabilitation. Yavuzer et al. [15] found that MT was beneficial for improving hand function in stroke patients, while Rothgangel et al. [14] reported through a systematic review that there was moderate evidence of MT improving upper limb function in stroke, phantom pain, and complex regional pain syndrome. MT incorporates a mirror to reflect the image of the unaffected limb in replacement of the image of the paretic limb to eliminate abnormal sensations and restore motor function [13,23].

Several studies have suggested explanations for the positive effects of MT. Altschuler et al. [25] suggested that visual input from a mirror replaces a portion of the missing proprioceptive input, and the illusions of the moving hand may increase recruitment of the premotor cortex through the connection of the visual area. Stevens and Stoykov [26], during their mirror training, noted that motor imagery of successful movement can be helpful for functional recovery. Deconinck et al. [27] explained the mechanisms underlying the positive effect from mirror visual feedback (MVF) with three hypotheses: first, MVF may activate crucial parts of the motor system related to the mirror neuron that can induce motor learning. The brain network of the premotor cortex, supplementary motor area, inferior frontal gyrus, and inferior parietal lobe, which are thought to be activated similarly during MVF and action observation, plays an important role in action recognition, motor learning, and rehabilitation [28,29]. Second, MVF may promote the recruitment of ipsilateral motor pathways toward the affected side. This may be especially beneficial because prolonged functional disability from stroke may induce the “learned non-use” and inhibition of the healthy hemisphere to the affected hemisphere [30,31,32,33,34]. Third, MVF may increase visual-spatial attention to the affected side [35]. Deconinck et Al. mentioned that training, such as MT, can activate a broad network of the brain, which contributes to attention and action monitoring [27]. With respect to the neuroplastic effects of MT, studies have reported an augmented activation of the affected side hemisphere associated with impaired movements with or without a decreased activation of the unaffected side [36,37,38,39].

Although many studies have reported the positive effects of MT [40,41,42], some randomized controlled trial studies have disputed with results of weak or no effects of MT compared to sham or conventional therapy. Thieme et al. [43] found no positive effect of MT on the sensorimotor function of the arm, neither in the activities of daily living, nor quality of life, except for visuospatial neglect. In addition, Antoniotti et al. [44] conducted an assessor-blinded randomized controlled trial to evaluate the recovery of the upper limb using MT and found no significant differences between actual and sham MT. Michielsen et al. [45] also found no evidence of increased activity areas belonging to the motor or mirror neuron system during MT. These limitations might either be due to the poor effect of MT itself or the lack of methodological detail. In fact, the most recent Cochrane review claimed that MT is effective; however, there remains uncertainty in the quality of evidence [13]. Among the limitations, poor reporting of methodological quality has been mentioned.

Some studies on MT have suggested the importance of attention during treatment. Colomer et al. [46] studied the effect of MT on light touch and explained the positive effect of MT on tactile sensitivity. They suggested that participants required special attention to be paid to the task during treatment and believed that this attention may have increased the activity of the somatosensory cortical cortex. Another study that compared individual and group MT showed weaker improvement in group MT [43]. The study explained that the underlying result may be due to poor attention during group therapy.

Of the many consequences of stroke, attention impairment is one of the most common cognitive impairments [6]. Attentional impairments are presented in a variety of ways, including a reduction in concentration and error control, distraction, difficulties completing more than one task at a time, mental slowness, and fatigability [6]. Cognitive productivity can be reduced when attention is impaired, even with other cognitive functions intact, and can play a key role in learning motor skills. Therefore, the efficiency of treatment that requires motor learning skills may be reduced with impaired attention [47]. On this basis, stroke patients with attention deficits may not reap the full benefit from MT. In fact, an estimated 46% to 92% of acute stroke patients present attention deficits [48,49]; therefore, many of the patients can be considered as attending therapy sessions without full attention on the mirror. However, as far as our knowledge, there are no studies that have investigated how much attention patients paid to the mirror during MT.

In a previous study [50] assessing the attention of three participants during MT, participants showed tendencies of distraction and gazed at the unaffected arm instead of the mirror. Consequently, a unilateral MT method [51,52] was devised to avoid gazing toward the unaffected side to make a clear difference. Therefore, this study aims to compare the duration and frequency of stroke patients looking at mirrors during two different methods of MT to confirm how much patients look at the mirror during MT, and suggest improvements for clinicians and future studies.

## 2. Materials and Methods

### 2.1. Participants

All sixteen participants included in the present study were recruited from the inpatient unit of “N” hospital located in Gyeonggi-do, Republic of Korea. The purpose and procedures of the study were explained to every participant. The inclusion criteria for participants were those who had suffered a stroke with hemiplegia, were subacute to chronic stages at least 3 months since the onset, could understand and follow instructions, scored over 21 points on a Korean version of the Mini Mental State Examination (K-MMSE), and had a Fugl–Meyer score for upper extremity (FMA-UE) between 26–56, and presented with mild-to-moderate motor impairment. The exclusion criteria were patients with dementia or other mental disorders, those who presented with hemiplegic neglect or apraxia, and those with musculoskeletal disorders in the upper extremities. Among all the participants, those who met the inclusion criteria, understood the study, and provided consent were included. This study was approved by the Sahmyook University Institutional Review Board (2-1040781-AB-N-01-2017074HR).

### 2.2. Procedure

The participants engaged in two treatment sessions of 30 min mirror therapy with a 1 week interval between the sessions. In the first week, each of them was randomly assigned to either conventional bilateral mirror therapy (BMT) or unilateral mirror therapy (UMT) using a screen. Then, in the next week, they were assigned to the other treatment depending on their first treatment protocol. The filming of the unaffected side was preceded prior to all treatment sessions. They were filmed during the treatments, and their gaze directions and preparatory actions were analyzed using behavioral coding software (Observer XT, Noldus, The Netherlands). From the footage, the time and number of preparations, distractions, and mirror gazes were recorded and analyzed using the software.

### 2.3. Intervention

#### 2.3.1. Protocol for the BMT

**Figure 1:**
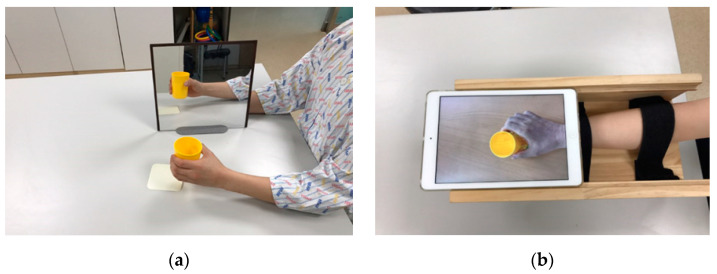
Protocols for two different MT methods: (**a**) BMT and (**b**) UMT.

A 30 cm × 30 cm mirror was used during the treatment. Participants sat on either a wheelchair or a chair, and the mirror was placed vertically in front. The unaffected upper limb was placed in front of the mirror so that the reflected illusion of the limb could be hallucinated to represent the affected upper limb. The treatment session included the following 11 tasks: wiping the table, pushing the arm forward, moving an object along the table, flipping an object, raising and then lowering a cup, grabbing objects of different sizes, turning a page, inserting a coin into a piggy bank, lifting a “Go ball” on the palm, picking up a clip, and inserting a nail into a hole (Figure 1a).

#### 2.3.2. Protocol for the UMT

A tablet (iPad Air 1, Apple, Cupertino, CA, USA.) was affixed to a box using Velcro fasteners, and four wheels were attached to the bottom. Participants inserted their affected arm into the box. Before the treatment, all 11 tasks used in the BMT were recorded while each participant performed the tasks with their unaffected arm. Then, the footage was left–right reversed to display them on the tablet screen as the mirror illusion. During the treatment, participants performed the same tasks as BMT, while they watched the recorded footage of their own unaffected arms. The unaffected arms remained resting and hidden under the desk. The method used for UMT was based on previous studies [51,52], which also used a movable box with a screen attached on top to display the filmed movement of the unaffected hand. UMT was used to reduce postural asymmetry and provide reciprocal training (Figure 1b).

### 2.4. Data Analysis

#### 2.4.1. Outcome Measures

The outcome measures in this study consisted of three different main parameters: total gaze duration, total gaze bouts, and average gaze duration per bout. In the total gaze duration, the sub-parameters included the total mirror gaze duration (TGD), which measured the sum of the time in seconds that participants gazed toward the mirror during one session of MT; total distracted duration (TDD), which measured the sum of the time in seconds that participants gazed elsewhere (other than the mirror) during one session of MT; and total preparatory duration (TPD), which measured the sum of the time in seconds that participants spent to prepare a task during one session of MT.

The sub-parameters for total gaze bouts included the total mirror gaze bouts (TGB), which measured the sum of the bouts in numbers that participants gazed in the mirror during one session of MT; total distracted bouts (TDB), which measured the sum of the bouts in numbers that participants gazed elsewhere (other than the mirror) during one session of MT; and total preparatory bouts (TPB), which measured the sum of the bouts in numbers that participants spent to prepare a task during one session of MT.

The average gaze duration per bout was calculated by dividing the total duration by the total number of bouts. The sub-parameters for the average gaze duration included the average mirror gaze duration per bout (AGD), which measured the average time of gazing in the mirror in one bout; average distracted duration per bout (ADB), which measured the average time of distraction per one bout; and average preparatory duration per bout (APB), which measured the average time of preparation per one bout. We assumed that the mirror gaze duration was a meaningful treatment duration, indicating that participants had actually paid attention during the treatment.

#### 2.4.2. Data Analysis

Statistical analysis of all data was performed using SPSS (version 19.0, IBM Corp., Armonk, NY, USA.). The Kolmogorov–Smirnov test was used to evaluate normality. A paired *t*-test was used to compare the results of each measure between the BMT and UMT. The statistical significance was set at *p* < 0.05.

#### 2.4.3. Sample Size

The sample size was determined using G*Power software (version 3.1.9.7, 2020, Heinrich Heine University Düsseldorf, Düsseldorf, Germany) with the alpha error of probability and power set at 0.05 and 0.8, respectively. The effect size was set at 0.80, based on the previous study [50]. Accordingly, a sample size of 15 participants was determined to be necessary. Finally, a total of sixteen participants were recruited.

## 3. Results

Sixteen patients who participated in this study received random orders of the two different MT methods: BMT and UMT. The general characteristics of the participants are presented in Table 1.

**Figure 2:**
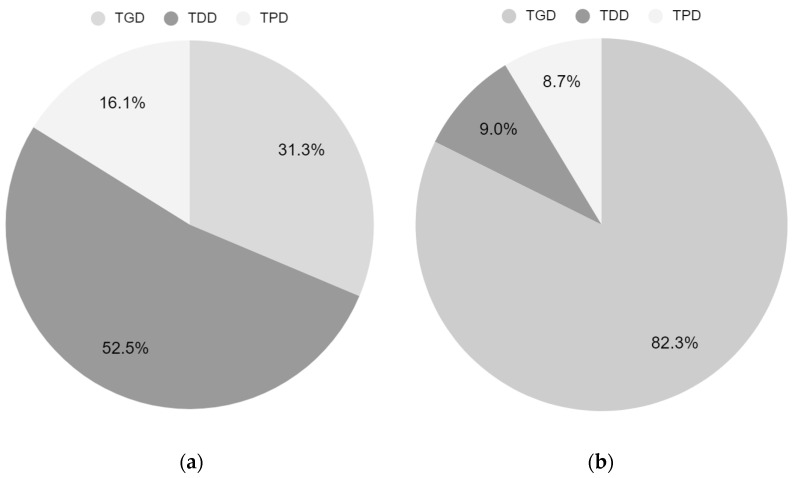
Percentage of total mirror gaze duration (TGD), total distracted duration (TDD), and total preparatory duration (TPD); (**a**) BMT and (**b**) UMT.

The results of behavioral analysis of the participants are shown in Table 2. In a comparison of the duration, all the variables showed significant differences (*p* < 0.05) between the two methods. TGD was significantly increased in UMT, whereas TDD and TPD were significantly lower (*p* < 0.001) in UMT. The TGD in BMT was 564.04 s but increased to 1482.45 s. TDD was 945.61 s in BMT, while 162.03 s in UMT, suggesting a significant lower time of distraction (*p* < 0.001). TPD was 290.35 s in BMT and 155.53 s in UMT, also showing a significant lower duration (*p* < 0.05). In the 30 min treatment session, TGD was 31.3% of the total duration in BMT, while it was 82.3% in UMT (Figure 2).

In comparing the number of bouts, TGB did not show significant differences between the two methods; however, there were significant decreases in bouts in TDB (*p* < 0.05) and TPB (*p* < 0.005). TDB was 129.23 in BMT while 101.23 in UMT. TPB was 18.42 in BMT while 9.56 in UMT. Although it did not reach statistical significance in TGB, the number of alterations of the gaze from mirror to elsewhere decreased in UMT compared to BMT.

The calculated average duration per bout showed significant differences (*p* < 0.005) in AGD and ADP, whereas APB did not. The AGD in BMT was 5.52 s while 21.81 s in UMT. ADB was 9.22 s in BMT while 3.00 s in UMT. The average mirror gaze duration was approximately four times longer in UMT than in BMT.

## 4. Discussion

The present study compared two different methods used in MT to assess the attention of participants during MT. To the best of our knowledge, this study is the first attempt to measure the attention to the mirror of stroke patients during MT. According to the most recent Cochrane review, while MT has been widely used as a rehabilitation strategy for stroke patients and its effectiveness is known to be well accepted, some uncertainty remains [13]. The review found moderate-quality evidence to support the effectiveness of MT in improving motor function, impairment, and ADL, while some studies suggested only poor effectiveness of MT on motor function and uncertain activity of mirror-related brain areas [44,45,46,53]. If the causes for the poor effectiveness can be improved, the results of MT can be enhanced; however, there are no clear interpretations to explain the poor results of MT. Our hypothesis prior to the experiment was that attention, that is, the ability of stroke patients to maintain focus on the mirror during MT, might be a contributing factor in altering the effectiveness of the treatment.

In the previous study [50], three participants showed distraction during MT and presented poor gaze or focus on the mirror. Participants did not present any neglect; however, they were easily distracted toward their unaffected arms. The present study showed similar results in conventional BMT using a mirror. During the single 30 min treatment session of BMT, they were only able to focus on the mirror for 31.3% of the total duration. Except for preparations, distracted durations consumed about 52.5% of the duration, suggesting that for more than half of the time, they might be poorly treated. To eliminate the possible distractions of looking toward unaffected arms, UMT using a screen device was used. The normal movements of the unaffected arms were recorded prior to the treatment and displayed on the screen with the left–right reversed during the treatment to represent mirror illusions of the affected arms. The results from UMT were significant as mirror gaze duration and average duration per bout during UMT increased considerably. In UMT, participants remained gazing towards the screen for 82.3% of the total duration, nearly tripling the duration of attention. In addition, the distracted duration decreased to 9.0%, suggesting that considerable attention had been maintained during the treatment. Although it was not statistically significant, the number of total bouts of looking toward the mirror and somewhere else other than the mirror was reduced. This suggests that the alteration of gazes decreased and the participants maintained gazing towards the mirror. AGD, which measures the average duration of mirror gaze maintained from when the participants start to gaze towards the mirror until they are distracted, was considerably increased in UMT. This result also implies that the participant’s attention was maintained longer in UMT.

Attention deficits are very common after stroke and can be a major contributing factor for motor learning and quality of life after 6 months of onset [47,54]. Attention and distractibility are also associated with functional impairment [48]. Previous studies mentioned the possible influences of maintaining attention during treatment. Few studies have outlined that conventional MT requires a high concentration of participants. Colomer et al. [46] reported that the participants were required to pay special attention during MT, which contributed to the improvement in tactile sensitivity. Thieme et al. [43] found difficulties in group MT as participants were challenged with maintaining attention during MT. Cristina et al. [55] stated that attention and complexity of MT tasks are crucial factors in inducing long-term neuroplasticity. However, to the best of our knowledge, no studies have considered the effect of attention on MT.

Studies that investigated MT have outlined the importance of controlling the participants and assessors, and inadequate results might be due to bias from them [43,44,56]. However, the results of this study imply the importance of controlling methodology. Conventional MT places unaffected limbs in front of the mirror to create an illusion [25]; however, this can cause patients to become easily distracted away from the mirror, as shown in the results of this study. Thus, the unaffected arm was hidden away from sight to improve attention toward the mirrored illusion. This procedure eventually created unilateral training of the hemiplegic side. We believe that clinicians and future studies should consider not only which treatment method would be beneficial, but also how the method can be delivered most effectively to patients.

This study has several limitations. First, the direct effects of MT on participants were not considered. Although this study examined which method of the two induced stronger attention from participants, it does not imply that one method is more effective than the other. The participants’ attentional abilities may benefit from longer treatment sessions of MT. Therefore, a further controlled trial should be conducted to compare the results thoroughly. Second, this study contained only two sessions of BMT and UMT with a small number of sample size, thus, the results cannot be extrapolated for the chronic post-stroke rehabilitation since post-stroke survivors require more repetitive daily training. Finally, this study only observed the behaviors of the participants to compare the duration and bouts and not the actual influence of the methods on brain activity. Future studies may explore the relationships between different MT methods and results of psychological and cognitive assessment, and/or compare brain activities related to attention during or after the two methods of BMT and UMT using a functional magnetic resonance imaging.

## 5. Conclusions

This study compared two different methods of MT, BMT and UMT, to observe the attention of participants during treatment. During the conventional BMT, participants remained attentive for only one-third of the treatment sessions, while they maintained a focus nearly three times longer in UMT. UMT may be an alternative option for MT to improve participants’ attention toward treatment.

## Figures and Tables

**Table 1:** General characteristics of the participants.

Characteristics	P1	P2	P3	P4	P5	P6	P7	P8	P9	P10	P11	P12	P13	P14	P15	P16	Mean
Age (years)	58	66	49	65	58	64	63	52	57	54	65	67	53	58	64	66	59.94
Sex	M	F	F	F	M	M	F	M	M	M	M	F	M	M	M	M	M 11/F 5
Height (cm)	171	162	173	157	169	169	160	158	160	173	165	154	168	168	172	162	165.06
Weight (kg)	71	62	64	63	58	66	61	64	65	72	68	63	64	68	66	64	64.94
Onset time of stroke (month)	9	3	11	6	5	4	6	10	5	5	10	11	5	5	10	5	6.88
Types of stroke	IN	IN	HE	IN	HE	IN	IN	HE	IN	HE	HE	IN	IN	HE	IN	IN	IN 10/HE 6
Affected side	L	L	R	L	R	L	L	R	L	L	R	R	L	L	R	L	L 10/R 6
MMSE	26	24	28	29	24	26	28	24	30	29	24	25	27	26	25	30	26.56

Abbreviations: P, participant; M, male; F, female; IN, infarction; HE, hemorrhage; L, left; R, right; and MMSE, mini-mental state examination.

**Table 2:** Results of Behavioral Analysis of the Participants.

Variables.	BMT	UMT	*t*	*p*
TGD (seconds)	564.04 ± 365.27	1482.45 ± 123.81	−10.079	0.000
TDD (seconds)	945.61 ± 119.58	162.03 ± 86.53	8.347	0.000
TPD (seconds)	290.35 ± 80.89	155.53 ± 64.15	3.307	0.005
TGB (numbers)	129.23 ± 76.40	101.13 ± 65.43	1.120	0.280
TDB (numbers)	136.45 ± 7.60	73.38 ± 6.33	2.457	0.027
TPB (numbers)	18.42 ± 19.45	9.56 ± 10.25	4.184	0.001
AGD (seconds)	5.52 ± 5.57	21.81 ± 14.33	−4.142	0.001
ADB (seconds)	9.22 ± 6.77	3.00 ± 2.72	3.569	0.003
APB (seconds)	16.26 ± 6.48	22.84 ± 20.08	−0.160	0.875

Abbreviations: BMT, bilateral mirror therapy; UMT, unilateral mirror therapy; TGD, total mirror gaze duration; TDD, total distracted duration; TPD, total preparatory duration; TGB, total mirror gaze bouts; TDB, total distracted bouts; TPB, total preparatory bouts; AGD, average mirror gaze duration per bout; ADB, average distracted duration per bout; and APB, average preparatory duration per bout. Values are expressed as the mean ± standard deviation.

## Data Availability

The data presented in this study are available on request from the corresponding author.
